# ACE2‐EPC‐EXs protect ageing ECs against hypoxia/reoxygenation‐induced injury through the miR‐18a/Nox2/ROS pathway

**DOI:** 10.1111/jcmm.13471

**Published:** 2018-01-24

**Authors:** Cheng Zhang, Jinju Wang, Xiaotang Ma, Wenjun Wang, Bin Zhao, Yanfang Chen, Can Chen, Ji C. Bihl

**Affiliations:** ^1^ Department of Pharmacology & Toxicology Boonshoft School of Medicine Wright State University Dayton OH USA; ^2^ Institute of Spinal Surgery and Neurology The First Affiliated Hospital of South China University Hengyang China; ^3^ Guangdong Key Laboratory of Age‐related Cardiac and Cerebral Diseases The Affiliated Hospital of Guangdong Medical University Zhanjiang Guangdong China

**Keywords:** ACE2‐EPCs‐EXs, miR‐18a, oxidative stress, ECs, ageing

## Abstract

Oxidative stress is one of the mechanisms of ageing‐associated vascular dysfunction. Angiotensin‐converting enzyme 2 (ACE2) and microRNA (miR)‐18a have shown to be down‐regulated in ageing cells. Our previous study has shown that ACE2‐primed endothelial progenitor cells (ACE2‐EPCs) have protective effects on endothelial cells (ECs), which might be due to their released exosomes (EXs). Here, we aimed to investigate whether ACE2‐EPC‐EXs could attenuate hypoxia/reoxygenation (H/R)‐induced injury in ageing ECs through their carried miR‐18a. Young and angiotensin II‐induced ageing ECs were subjected to H/R and co‐cultured with vehicle (medium), EPC‐EXs, ACE2‐EPCs‐EXs, ACE2‐EPCs‐EXs + DX600 or ACE2‐EPCs‐EXs with miR‐18a deficiency (ACE2‐EPCs‐EXs^anti‐miR‐18a^). Results showed (1) ageing ECs displayed increased senescence, apoptosis and ROS production, but decreased ACE2 and miR‐18a expressions and tube formation ability; (2) under H/R condition, ageing ECs showed higher rate of apoptosis, ROS overproduction and nitric oxide reduction, up‐regulation of Nox2, down‐regulation of ACE2, miR‐18a and eNOS, and compromised tube formation ability; (3) compared with EPC‐EXs, ACE2‐EPC‐EXs had better efficiencies on protecting ECs from H/R‐induced changes; (4) The protective effects were less seen in ACE2‐EPCs‐EXs + DX600 and ACE2‐EPCs‐EXs^anti‐miR‐18a^ groups. These data suggest that ACE‐EPCs‐EXs have better protective effects on H/R injury in ageing ECs which could be through their carried miR‐18a and subsequently down‐regulating the Nox2/ROS pathway.

## Introduction

Oxidative stress has been recognized as a main factor accounting for endothelial/vascular dysfunction, which has been observed in many cardio‐ and cerebrovascular diseases 1. For example, increased production of reactive oxygen species (ROS) has been linked to impaired endothelial vasomotor function in cardiovascular diseases 2, 3, 4. We have reported that the ROS production is increased in hypoxia‐treated endothelial cells (ECs) and oxygen and glucose deprivation brain slice models 5, 6. Moreover, the oxidative stress is involved in ageing‐associated vascular dysfunction 7. As we know, the vascular diseases are prone to occur in elders with increased oxidative stress. Therefore, attenuating of oxidative stress is a very important strategy to improve vascular function and subsequently to treat vascular disease, especially in elders.

Angiotensin‐converting enzyme 2 (ACE2), a negative regulator of renin–angiotensin system through converting angiotensin II (Ang II) to angiotensin (1–7), is known to participate in the regulation of vascular functions 8, 9. The ACE2/angiotensin (1–7) signalling has been shown to prevent vascular dysfunction and oxidative stress 10. Our group has demonstrated that neuronal overexpression of ACE2 protected cerebral microvasculartures from ischaemia‐induced injury through modulating the NADPH oxidase (Nox)/ROS and endothelial nitric oxide synthase/nitric oxide (eNOS/NO) pathways 11. ACE2 deficiency increases oxidative stress and endothelial dysfunction in cerebral arteries of adult mice and augmented endothelial dysfunction during ageing 8. Later, we showed that neuronal overexpression of ACE2 reduces ischaemic cerebral injury, with a greater efficacy in ageing mice 6. Moreover, we found the ACE2 has protective effects on endothelial progenitor cells (EPCs). Overexpression of ACE2 on EPCs exhibited protective effects on EPCs by decreasing apoptosis, attenuating oxidative stress and improving EPC function 12. EPCs, which are bone marrow‐derived progenitor cell with the ability to differentiate to endothelial cells (ECs), play a critical role in maintaining endothelial function and haemostasis. We further demonstrated that ACE2‐primed EPCs (ACE2‐EPCs) offered a better therapeutic effect than EPCs on ischaemia‐injured brain by vasculature protection and angiogenesis promotion 12. This suggests that ACE2‐EPCs have vascular protection *via* anti‐oxidative effect, especially under ageing situation.

Exosomes (EXs; diameter: 30–120 nm) are one type of extracellular vesicles. They can mediate cell–cell communication *via* conveying their cargoes include proteins and microRNAs (miRs) 5. In recent years, increasing evidence shows that stem cell‐derived extracellular vesicles include EXs could convey the benefits of their parent cells. Indeed, inducible pluripotent stem cell‐derived EXs can protect cardiomyocytes against ischaemic injury through delivering miR‐21 13. EPCs‐derived extracellular vesicles can protect kidney against ischaemia/reperfusion injury 14 and improve the angiogenic effects in injured ECs associating with the delivery of miR‐126 5. Recently, our group demonstrated that EPCs‐derived vesicles from healthy controls have protective effects on the function of EPCs from patients with diabetes through their carried miR‐126 15. However, the role of ACE2‐EPC‐EXs in modulating the oxidative stress in ageing remains largely unknown. As we know, miRs play an important role in vascular ageing processes 16, 17. As one of the ageing‐related miRs, miR‐18a is found down‐regulated in human ageing 18. More interestingly, miR‐18a could regulate the proliferation and tube formation abilities of ECs and expresses in EPCs 19. As both of miR‐18a and ACE2 are down‐regulated in human ageing, exploring the role of miR‐18a in the function of ACE2‐EPC‐EXs deserves further investigation.

In this study, we aimed to investigate whether ACE2‐EPC‐EXs can exert better efficacy in protecting ageing ECs than young ECs against hypoxia/reoxygenation (H/R)‐induced oxidative stress through the miR‐18a/Nox2/ROS pathway.

## Materials and methods

### Cell culture

EPCs were isolated from bone marrow of adult C57BJ/6 mice, as we previously described 12. Brain ECs were purchased from Cell systems (Kirkland, WA, USA). Passages 6–8 of ECs were used as young ECs. To induce ageing EC model, cells were maintained in serum‐free medium for 12 hrs in the CSC culture medium (Cell systems) to ensure G0 arrest, then angiotensin II (Ang II, 10^−6^ M; Sigma‐Aldrich, St. Louis, MO, USA) was added into the culture medium and incubated for 48 hrs 20. The EC ageing model was characterized using β‐gal staining and evaluation of p16 expression.

### EPCs transfection

To overexpress ACE2, EPCs were transfected with lenti‐GFP or lenti‐ACE2 for 24 hrs as we previously reported 12. The efficiency of transfection was determined using a fluorescence microscope (EVOS, Thermo Fisher Scientific, Waltham, MA, USA). For blocking experiment, EPCs were incubated with ACE2 inhibitor (DX600, 1 μM) for 24 hrs after transduction with lenti‐ACE2. To down‐regulate miR‐18a in ACE2‐EPCs, cells (60–70% confluence) were transfected with miR‐18a inhibitors (10 nM; Dharmacon) or miR‐18a scramble control (sc) using lipofectamine 2000 Reagent (Invitrogen, Carlsbad, CA, USA) in serum‐free conditions for 6 hrs before changing to complete medium. Cells were collected 72 hrs after transfection to determine the transfection efficiency. The experiment was repeated six times. The EXs released from ACE2‐EPCs and ACE2‐EPCs transfected with miR‐18a inhibitors (ACE2‐EPCs^anti‐miR‐18a^) were used for EC co‐culture experiments.

### Collection of EXs released from EPCs

The protocol for collecting EXs from serum‐free conditional medium has been reported in our previous study 21. Briefly, EPCs, ACE2‐EPCs, ACE2‐EPCs^anti‐miR‐18a^ and ACE2‐EPCs^anti‐miR‐sc^ were cultured in serum‐free EPC basal medium supplemented with growth factors to release EXs which were denoted as EPC‐EXs, ACE2‐EPC‐EXs, ACE2‐EPC‐EXs ^anti‐miR‐sc^ or ACE2‐EPC‐EXs^anti‐miR‐18a^. After 24 hrs, the respective conditional medium was collected and centrifuged at 300 *g* for 15 min. to remove dead cells. The supernatants were centrifuged at 2000 *g* for 30 min. to remove cell debris, followed by centrifugation at 20,000 *g* for 70 min. and ultracentrifugation at 170,000 *g* for 90 min. to pellet EXs. The pelleted EXs were resuspended with phosphate‐buffered saline (PBS) and aliquoted for nanoparticle tracking analysis (NTA) and co‐culture experiments. PBS was filtered through 20‐nm filter (Whatman, Pittsburgh, PA, USA). To investigate the relationship of ACE2 and miR‐18a, DX600 was used to block the activity of ACE2.

### Nanoparticle tracking analysis of EPC‐EXs

The NanoSight NS300 (Malvern Instruments, Malvern, UK) was used to analyse the size and concentration of EXs at light‐scatter mode as we previously reported 21. Briefly, for size and concentration detection, the collected EXs were resuspended with 700 μl filtered PBS and analysed under light‐scatter mode of NTA. Three videos of typically 30 sec. duration were taken, with a frame rate of 30 frames per second. Data were analysed by NTA 3.0 software (Malvern Instruments) on a frame‐by‐frame basis. The experiment was repeated six times.

### H/R injury model of ECs

The young and ageing ECs injury model induced by H/R has been described in our previous study 5. Briefly, the young and ageing ECs were changed with fresh culture medium and cultured in a hypoxic chamber (1% O_2_, 5% CO_2_ and 94% N_2_; Biospherixhypoxia chamber, NY, USA) for 6 hrs, then reoxygenated by incubation in a standard 5% CO_2_ incubator for 24 hrs. Some plates of cells were harvested for senescence assay, quantitative real‐time PCR (qRT‐PCR) analysis, ROS production and tube formation assay. During reoxygenation, some plates of cells were co‐cultured with various types of ACE2‐EPC‐EXs. All experiments were repeated six times.

### Incorporation assay of ACE2‐EPC‐EXs with ECs

To determine whether ACE2‐EPC‐EXs can incorporate with ECs, the EPC‐EXs, ACE2‐EPC‐EXs and ACE2‐EPC‐EXs^anti‐miR‐18a^ were labelled with PKH 26 and co‐cultured with the young and ageing ECs 5. In brief, EXs were labelled with PKH 26 (2 × 10^−6^ M, Sigma‐Aldrich) for 5 min. at 37°C, followed by wash with 1× PBS and ultracentrifuged at 170,000 *g* for 90 min. After that, EXs (50 μg) were resuspended and added to the culture medium (1 ml) of ECs for co‐culture. After 24 hrs, ECs were washed with PBS for twice, and the nuclei of ECs were stained with DAPI (1 μg/ml, Wako Pure Chemical Industries Ltd, Richmond, VA, USA) for 2 min. Incorporation of EXs into ECs was observed by fluorescence microscopy (EVOS; Thermo Fisher Scientific). The experiment was repeated four times.

### Co‐culture of ACE2‐EPC‐EXs with ECs

To further elucidate whether ACE2‐EPC‐EXs can alter the function of ECs, young and ageing ECs were individually divided into four co‐culture groups: vehicle (co‐culture medium only), EPC‐EXs, ACE2‐EPC‐EXs, ACE2‐EPC‐EXs + DX600, ACE2‐EPC‐EXs^anti‐miR‐18a^. The work concentration of EXs (50 μg/ml) was determined based on our previous study 5. After 24 hrs, the mRNA level of ACE2 and miR‐18a level in ECs were analysed by quantitative RT‐PCR analysis. In addition, the apoptosis, ROS/NO production, Nox2/eNOS level and tube formation ability were determined. The experiment was repeated six times.

### Quantitative RT‐PCR analysis

After transfection, cells were washed with 1× PBS. The total mRNAs from EPCs and ECs were extracted using and lysed in Trizol (Thermo Fisher Scientific). The miRs from EPCs and ECs co‐cultured with different types of EPC‐EXs were extracted using mirVana miRNA Isolation kit (Qiagen, Hilden, Germany). To detect miR‐18a, reverse transcription (RT) reactions were performed using mirVana qRT‐PCR miRNA Detection Kit. The RT primer sequence for miR‐18a was 5′‐GTC GTA TCC AGT GCA GGG TCC GAG GTA TTC GCA CTG GATACG AC CTA TCT‐3′; the forward primer sequence for miR‐18a was 5′‐CAC GCA TAA GGT GCA TCT AGT GC‐3′; the reverse primer sequence for miR‐18a was 5′‐CCA GTG CAG GGT CCG AGG TA‐3. To measure the mRNA level of ACE2 and P16 in EPCs or ECs, RT was performed using the first strand cDNA synthesis kit (Qiagen) according to manufacturer's instruction. The forward primer of ACE2 was 5′‐AAGCTAGCATAGCCAGGTCCTCCTGGCTCCTTC‐3′; the reverse primer of ACE2 was 5′‐AAGTCGACCTAAAAGGAAGTCTGAGCATCATCACTG‐3′. The expression of U6 was used as endogenous control for each sample. The forward primer of U6 was 5′‐CTCGCTTCGGCAGCACA‐3′, the reverse primer of U6 was 5′‐AACGCTTCACGAATTTGCGT‐3′. Relative expression level of each gene was normalized to U6 and calculated using the 2^−ΔΔCT^ method. The experiment was repeated six times.

### Apoptosis assay

The apoptosis assay of young and ageing ECs was conducted using FITC Annexin V apoptosis detection kit (BD Biosciences, CA, USA) as previously described 5. In brief, cells were washed with PBS, resuspended with 100 μl 1× annexin‐binding buffer, incubated with 5 μl FITC‐conjugated Annexin V and 5 μl propidium iodide (PI) for 15 min. in the dark and then analysed by flow cytometry. The apoptotic cells were defined as Annexin V+/PI− cells. The experiment was repeated four times.

### Measurement of ROS generation

Intracellular ROS production was determined by dihydroethidium (DHE) (Sigma‐Aldrich) staining followed by flow cytometric analysis 5. Briefly, cells were incubated with 2 μM DHE solution at 37°C for 2 hrs, washed with PBS twice, trypsinized and centrifuged. The fluorescence intensity of cells was analysed by flow cytometry.

### Senescence assay

The senescence of ECs was determined by senescence‐associated β‐galactosidase (SA‐β‐gal) staining according to instruction of a commercial kit. Before staining, cell culture media was removed and the cells were rinsed with 1× PBS. Then, cells were fixed with fixative solution for 10–15 min. at room temperature and washed with 1× PBS for two times. Afterwards, 1 ml of the β‐gal staining solution was added to cells and incubated at 37°C overnight in a dry incubator (no CO_2_) to staining cells. After staining, cells were checked under a microscope (200× total magnification). The percentage of β‐gal‐positive cells (in blue colour) to the total numbers of cells was calculated.

### Tube formation assay for ECs

The tube formation assay was conducted using *in vitro* angiogenesis assay kit (Chemicon, Burlington, Massachusetts, USA). First, the EC matrix solution was thawed and mixed with the EC matrix diluent. Then, the EC matrix mixture was placed in a 96‐well tissue culture plate at 37°C for 1 hr to allow the matrix solution to solidify. ECs (1 × 10^4^ cells/well) were seeded onto the solidified matrix and incubated at regular cell culture conditions (5% CO_2_, 37°C). After 24 hrs post‐seeding, 2 μg/ml calcein (Fisher scientific) was directly added to the culture well and incubated for 20 min. prior to imaging under an inverted fluorescence microscope. Tubes were defined as a tube structure exhibiting a length four times of its width 22. Five random microscopic fields were assessed in each well. The average number of tubes per field was determined.

### Measurement of nitric oxide level

The membrane‐permeable indicator diaminofluorescein (DAF‐FM) diacetate (Invitrogen, Grand Island, NY, USA) was used to assess nitric oxide production 12.

Briefly, the ECs were incubated with 2 μM DAF‐FM diacetate in serum‐free CSC medium at 37°C for 30 min., washed with PBS twice, then incubated with CSC medium for 20 min. to allow complete de‐esterification of the intracellular diacetates. DAF‐FM fluorescence was measured using a spectrofluorometer.

### Western blot analysis

After 24 hrs co‐culture, proteins of ECs in different groups were extracted with cell lysis buffer (Thermo Fisher scientific) supplemented with complete mini protease inhibitor tablet (Roche, Basel, Switzerland). Then, the protein lysates were electrophoresed through SDS‐PAGE gel and transferred onto PVDF membranes. The membranes were blocked with 5% non‐fat milk for 1 hr at room temperature and incubated with primary antibody against Nox2 (1:1000; Santa Cruz), eNOS (1:1000; Abcam, Cambridge, United Kingdom) or β‐actin (1:4000; Sigma‐Aldrich) at 4°C overnight. On the next day, membranes were washed and incubated with horseradish‐peroxidase‐conjugated anti‐rabbit or antimouse IgG (1:40,000; Jackson Immuno Research Lab) for 1 hr at room temperature. Blots were developed with enhanced chemiluminescence developing solutions, and images were quantified under ImageJ software. The experiment was repeated six times.

### Statistical analysis

Data are expressed as mean ± S.E. Multiple comparisons were analysed by one‐ or two‐way ANOVA followed by a least significant distance (LSD) post hoc test. SPSS 23.0 statistical software was used for analysing the data. For all measurements, a *P* < 0.05 was considered statistic significant.

## Results

### Characterization of ageing ECs

**Figure 1:**
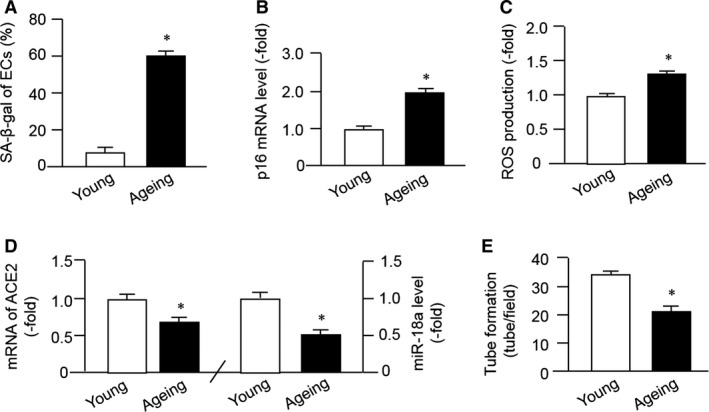
Characterization of ageing EC models induced by angiotensin II. (**A**–**B**) SA‐β‐gal staining and p16 expression. (**C**) ROS level. (**D**) The levels of ACE2 and miR18a. (**E**) Tube formation ability of ECs. *P < 0.05 *versus* young. Data are mean ± S.E.M. *n* = 4–6/group.

To assess the onset of senescence, we performed SA‐β‐gal staining, a biomarker for cellular senescence. The result showed that SA‐β‐gal staining was significantly increased in Ang II‐stimulated cells when compared to the control ECs (Fig. 1A), indicating the successful establishment of EC ageing model. We also found that the p16 level was significantly increased in ageing ECs (Fig. 1B). Of note, the levels of ACE2 and miR‐18a were decreased in ageing ECs (Fig. 1D). Ang II‐induced EC senescence leads to the ROS overproduction (Fig. 1C) and impairment of tube formation ability of ECs (Fig. 1E).

### ACE2 transfection increased the levels of ACE2 and miR‐18a in ACE2‐EPCs and their released EXs

**Figure 2:**
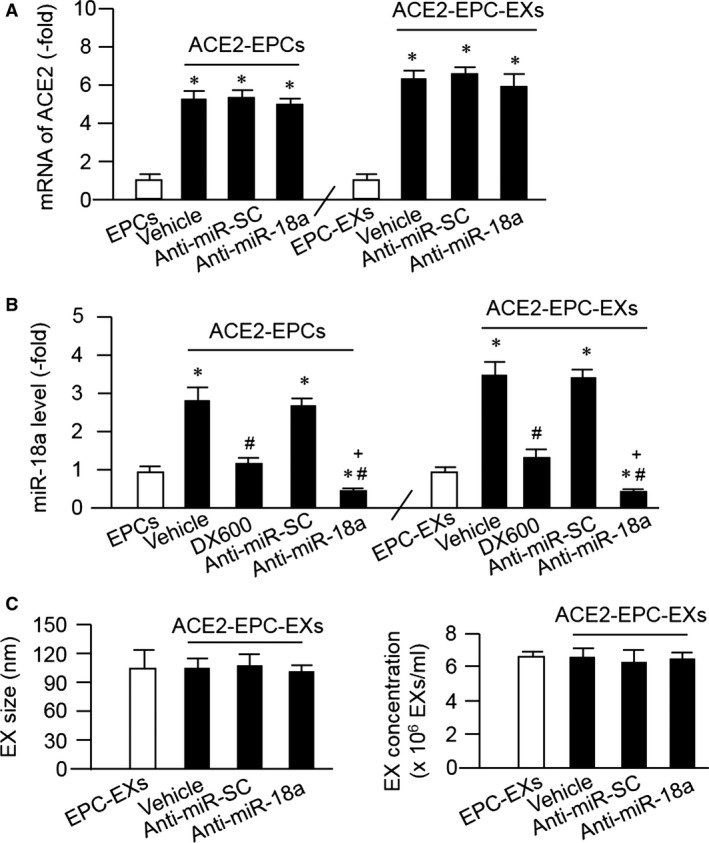
Analysis of the level of ACE2 and miR‐18a in ACE2‐EPCs and their released EXs, and assessment of EX size/concentration. (**A**–**B**) the mRNA level of ACE2 and the miR‐18a level in ACE2‐EPCs and the corresponding EXs. (**C**–**D**) summarized data of the size and concentration of the four types of EXs; *P < 0.05 *versus* EPCs or EPC‐EXs; ^#^ *P* < 0.05 *versus* vehicle; ^+^ *P* < 0.05 *versus* ACE2‐EPC‐EXs^anti‐miR‐sc^. Data are mean ± S.E.M. *n* = 6/group.

As shown in Figure 2A and B, the mRNA level of ACE2 was up‐regulated in ACE2‐EPCs, and ACE2‐EPCs released EXs as compared to EPCs and EPC‐EXs, indicating the successful generation of ACE2‐EPC‐EXs. Meanwhile, the miR‐18a level was increased in ACE2‐EPCs and ACE2‐EPC‐EXs, suggesting the potential role of miR‐18a in ACE2‐EPC‐EXs. Knock‐down of miR‐18a with inhibitors in ACE2‐EPCs did not change the expression of ACE2, but down‐regulated the miR‐18a level in ACE2‐EPCs and ACE‐EPC‐EXs. ACE2 inhibitor DX600 abolished ACE2 induced increased of miR‐18a in both ACE2‐EPCs and ACE2‐EPC‐EXs.

According to the NTA results (Fig. 2C and D), there were no significant differences of size and concentration among the four types of EXs, indicating miR‐18a inhibitor transfection did not change EX release of ACE2‐EPCs.

### ACE2‐EPC‐EX co‐culture increased the level of ACE2 and miR‐18a in ECs

**Figure 3:**
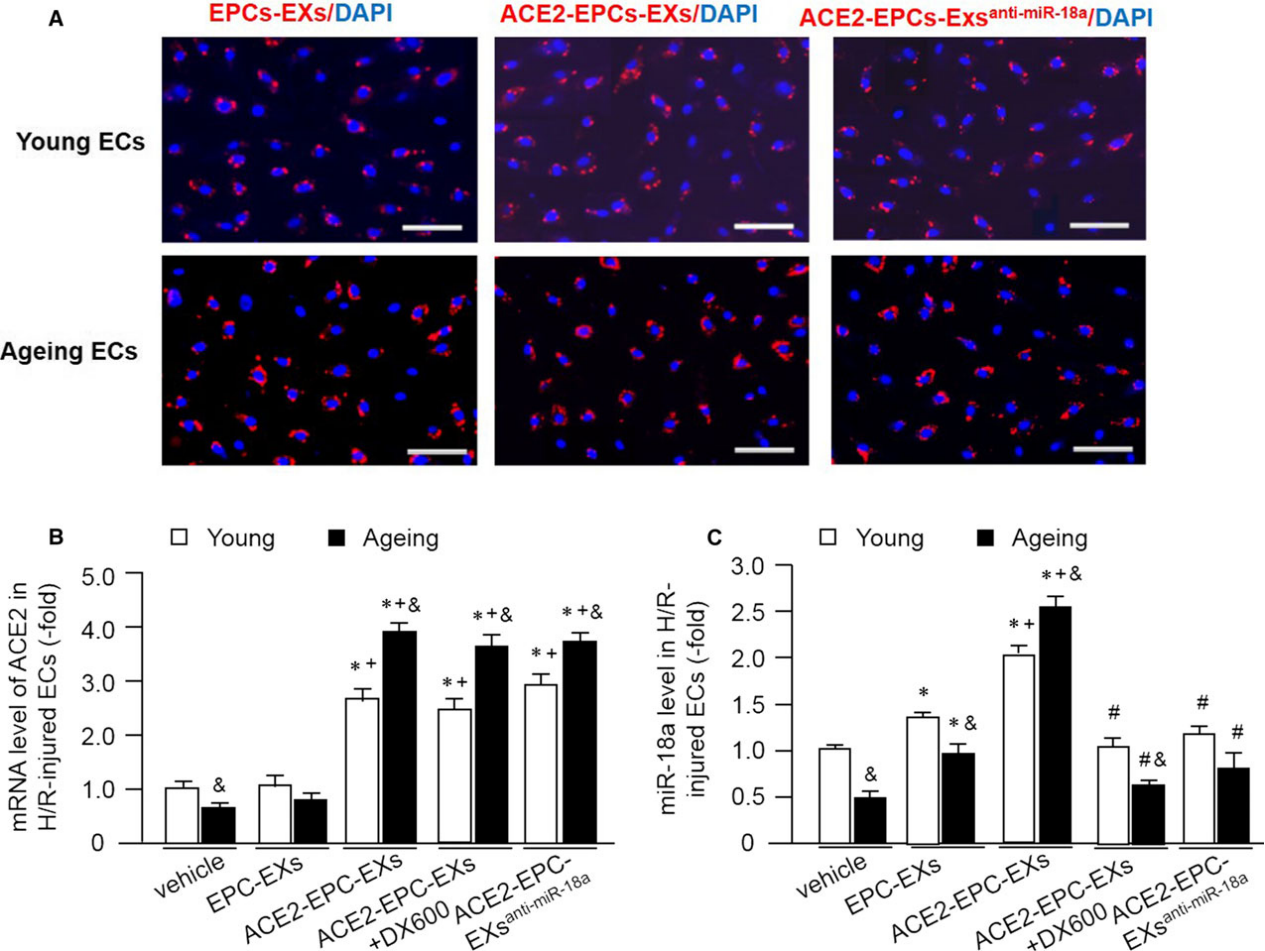
ACE2‐EPC‐EXs incorporated with H/R‐injured ECs and altered the levels of ACE2 and miR18a in ECs. (**A**) Various types of EPC‐EXs labelled with PKH26 were up‐taken by young and ageing ECs after 24‐hrs co‐culture; red: PKH26 labelled with EPC‐EXs; blue: nucleus counterstained with DAPI. Scale bar, 100 μm. (**B**) The mRNA level of ACE2 in ECs after co‐incubations with different EPC‐EXs. (**C**) The level of miR‐18a in ECs after co‐incubation with different EPC‐EXs. *P < 0.05 *versus* vehicle; ^+^ *P* < 0.05 *versus* EPC‐EXs; ^#^ *P* < 0.05 *versus* ACE2‐EPC‐EXs; ^&^ *P* < 0.05 *versus* young. Data are expressed as mean ± S.E.M., *n* = 4/group.

As shown in Figure 3A, EXs labelled with PKH26 were observed in the cytoplasm of both young and ageing ECs, indicating both young and ageing ECs could uptake EXs. To elucidate whether ACE‐EPC‐EXs harbouring ACE2 and miR‐18a can transfer its cargoes to ECs, we performed quantitative RT‐PCR to detect the level of ACE2 and miR‐18a in young and ageing ECs after co‐cultured with three different types of EPC‐EXs. Our data (Fig. 3B) showed that there was no difference of ACE2 mRNA level in ECs between the vehicle and EPC‐EXs, whereas, ACE2‐EPC‐EXs co‐culture significantly increased the ACE2 level, with a higher level in ageing ECs. ACE2‐EPC‐EXs co‐culture was more effective than EPC‐EXs in increasing the miR‐18a level in ECs, with a higher level in ageing ECs. ACE2‐EPC‐EXs + DX600 or ACE2‐EPC‐EXs^anti‐miR‐18a^ co‐culture down‐regulated the miR‐18a level, but did not change the expression of ACE2 in ECs (Fig. 3C).

### ACE2‐EPC‐EXs were more effective than EPC‐EXs in decreasing H/R‐induced apoptosis and dysfunction in ECs, with a better effect in ageing ECs

**Figure 4:**
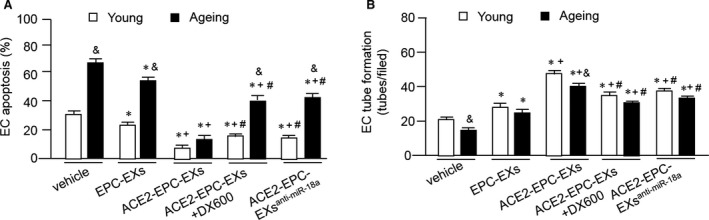
ACE2‐EPC‐EXs had better efficacy in decreasing apoptosis and improving the function of ECs in young and ageing ECs injured by H/R, with better efficacy in ageing ECs. (**A**) Apoptotic rate. (**B**) Tube formation of ECs. *P < 0.05 *versus* vehicle; ^+^ *P* < 0.05 *versus* EPC‐EXs; ^#^ *P* < 0.05 *versus* ACE2‐EPC‐EXs; ^&^ *P* < 0.05 *versus* young. Data are expressed as mean ± S.E.M., *n* = 4–6/group.

To determine whether ACE‐EPC‐EXs could protect ECs against H/R injury, we assessed the apoptotic rate of the young and ageing ECs co‐cultured with different types of EPC‐EXs. As shown in Figure 4A, H/R induced a higher apoptotic rate of ageing ECs than that of young ECs. EPC‐EXs co‐culture significantly decreased the apoptosis of young and ageing ECs as compared to that of vehicle (cultured in H/R condition without EX co‐culture). ACE2‐EPC‐EXs were more effective than EPC‐EXs in decreasing H/R‐induced EC apoptosis. More important, ACE2‐EPC‐EXs co‐culture decreased H/R‐induced apoptotic rate from 30 ± 3% to 9 ± 3% in young ECs and from 68 ± 4% to 17 ± 2% in ageing ECs. This data reflect that ACE2‐EPC‐EXs have a better anti‐apoptotic effect on ageing ECs than that on young ECs. Meanwhile, we found that knock‐down of miR‐18a could partially inhibit the anti‐apoptotic effect of ACE2‐EPC‐EXs. Similar effect was found in ECs co‐cultured with ACE2‐EPC‐EXs + DX600.

As shown in Figure 4B, the angiogenesis assay results showed that H/R impaired the tube formation ability of both young and ageing ECs, which could be improved by EPC‐EXs. ACE2‐EPC‐EXs were more effective than EPC‐EXs in increasing H/R‐induced impairment of EC tube formation ability. What is more, ACE2‐EPC‐EXs remarkably increased the tube formation ability of ECs by approximately 140% in young and 150% in ageing ones. ACE2‐EPC‐EXs + DX600 and ACE2‐EPC‐EXs^anti‐miR‐18a^ partially decreased the pro‐angiogenic effect of ACE2‐EPC‐EXs, suggesting miR‐18a plays a role in the pro‐angiogenic effect of ACE2‐EPC‐EXs.

### ACE2‐EPC‐EXs were more effective than EPC‐EXs in decreasing H/R‐induced ROS overproduction and Nox2 expression in ECs, with a better effect in ageing ECs

**Figure 5:**
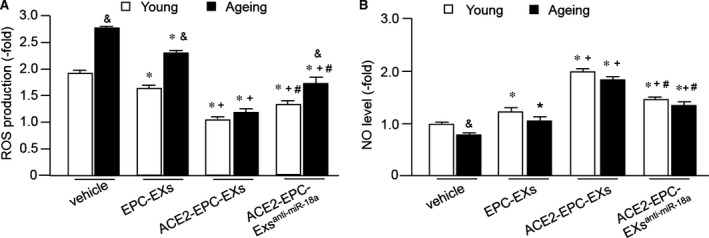
ACE2‐EPC‐EXs had better efficacy in decreasing ROS overproduction and increasing nitric oxide down‐regulation induced by H/R in young and ageing ECs, with better efficacy in ageing ECs. (**A**) ROS level. (**B**) Nitric oxide level. *P < 0.05 *versus* vehicle; ^+^ *P* < 0.05 *versus* EPC‐EXs; ^#^ *P* < 0.05 *versus* ACE2‐EPC‐EXs; ^&^ *P* < 0.05 *versus* young. Data are expressed as mean ± S.E.M., *n* = 6/group.

To determine whether ACE2‐EPC‐EXs could protect ECs against H/R injury by attenuating the oxidative stress, we analysed ROS production of the young and ageing ECs co‐cultured with different types of EPC‐EXs. As shown in Figure 5A, H/R induced a high level of ROS in ageing ECs than the young ECs. EPC‐EXs co‐culture decreased ROS overproduction in both young and ageing ECs. ACE2‐EPC‐EXs were more effective than EPC‐EXs in decreasing H/R‐induced ROS overproduction. More important, ACE2‐EPC‐EXs co‐culture decreased H/R‐induced ROS overproduction by approximately 38% in young ECs and 55% in ageing ECs, which indicates ACE2‐EPC‐EXs exhibited a better anti‐oxidative effect in ageing ECs as compared to that of young ECs. Knock‐down of miR‐18a partially blocked the anti‐oxidative effect of ACE2‐EPC‐EXs in both types of ECs, with more inhibiting effects on ageing ECs, suggesting that miR‐18a might play a critical role in the anti‐oxidative effect of ACE2‐EPC‐EXs.

**Figure 6:**
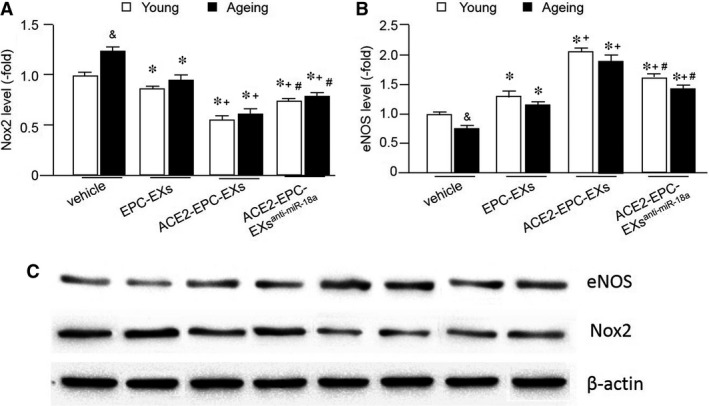
ACE2‐EPC‐EXs had better efficacy in decreasing the levels of Nox2 and increasing the level of eNOS in young and ageing ECs under H/R, with better efficacy in ageing ECs. (**A**) Nox2 level in ECs. (**B**) eNOS level in ECs. (**C**) Representative band for Nox2 and eNOS. *P < 0.05 *versus* vehicle; ^+^ *P* < 0.05 *versus* EPC‐EXs; ^#^ *P* < 0.05 *versus* ACE2‐EPC‐EXs; ^&^ *P* < 0.05 *versus* young. Data are expressed as mean ± S.E.M., *n* = 6/group.

As shown in Figure 6A, H/R induced a higher level of Nox2 in ageing ECs than the young ECs. Similarly, ACE2‐EPC‐EXs were more effective than EPC‐EXs in decreasing the level of Nox2. More important, ACE2‐EPC‐EXs co‐culture decreased H/R‐induced Nox2 level by approximately 40% in young ECs and 50% in ageing ECs, indicating ACE2‐EPC‐EXs offered a better effect in attenuating Nox2 level in ageing ECs as compared to that of young ECs. Knock‐down of miR‐18a partially blocked the effect of ACE2‐EPC‐EXs on decreasing Nox2 level in both types of ECs, suggesting that miR‐18a play a role of anti‐oxidative stress on ageing ECs through inhibiting Nox2.

### ACE2‐EPC‐EXs were more effective than EPC‐EXs in elevating H/R‐induced decrease in nitric oxide production and eNOS expression in ECs, with a better effect in ageing ECs

We also analysed nitric oxide production and eNOS level of the young and ageing ECs co‐cultured with different types of EPC‐EXs. As shown in Figure 5B, H/R decreased the level of nitric oxide in ageing ECs than the young ECs. EPC‐EXs co‐culture increased nitric oxide production in both young and ageing ECs. ACE2‐EPC‐EXs were more effective than EPC‐EXs in increasing the production of nitric oxide. Moreover, ACE2‐EPC‐EXs co‐culture increased nitric oxide production by approximately 81% in young ECs and 91% in ageing ECs, which indicates ACE2‐EPC‐EXs exhibited a better effect in ageing ECs as compared to that in young ECs. Knock‐down of miR‐18a partially blocked this effect of ACE2‐EPC‐EXs in both types of ECs, with more inhibiting effects on ageing ECs.

As shown in Figure 6B, H/R decreased the level of eNOS in ageing ECs than the young ECs. EPC‐EXs co‐culture significantly increased eNOS level in both young and ageing ECs. ACE2‐EPC‐EXs co‐culture increased the eNOS level by approximately 143% in young ECs and 166% in ageing ECs, indicating ACE2‐EPC‐EXs offered a better effect on augmenting eNOS level in ageing ECs as compared to that on young ECs. Similarly, knock‐down of miR‐18a partially blocked this effect. Altogether, ACE2‐EPC‐EXs could improve H/R‐induced EC function through miR‐18a/eNOS/NO pathway.

## Discussion

Oxidative stress is one of the major mechanisms involved in vascular dysfunction 23. Previous studies have revealed that vascular oxidative stress develops with ageing as a result of increased production of ROS, in the face of unchanged or reduced antioxidant defences 24. Therefore, decreasing ROS overproduction might be able to improve ageing‐associated vascular dysfunction. In the present study, we produced an ageing EC model using Ang II as previously reported 20. Ang II‐stimulated ECs had an elevated p16 level and increased percentage of SA‐β‐gal‐positive cells suggesting cellular senescence along with compromised tube formation ability, indicating the success of the EC ageing model. These observations are supported by several studies showing that ageing is associated with an attenuated capacity of the endothelium 25, 26. Our data also showed that Ang II‐induced ageing ECs were much susceptible to H/R injury than young ECs as revealed by the increased apoptosis, ROS and Nox2 production, decreased nitric oxide and eNOS level along with the impairment of tube formation ability. This agrees with a previous study showing that senescent ECs are more prone to pro‐apoptotic stimuli which are largely due to a decreased nitric oxide production 27. Meanwhile, several studies have also reported that eNOS expression and nitric oxide production are declined with age 4, 28, 29.

We have previously reported that transplantation of EPCs has therapeutic effects on ischaemic stroke 12. In recent years, increasing evidence shows that stem cell‐derived extracellular vesicles include EXs could convey the benefits of their parent cells 30. In the present study, we demonstrated that EPC‐EXs elicit anti‐oxidative and anti‐apoptosis as evidenced by protecting ECs from H/R‐induced injury, which is associated with decreased ROS/Nox2 and increased NO/eNOS levels. However, we also found that the levels of miR‐18a and ACE2 are decreased in Ang II‐induced ageing ECs. Our group has previously demonstrated that ACE2‐EPCs have better efficacy than EPCs in protecting brain against ischaemia injury and promoting angiogenesis 12. Furthermore, we found that neuron overexpression of ACE2 protects cells against oxygen–glucose deprived‐induced injury, which is correlated with changes in Nox2/Nox4 expression and ROS production 31. Therefore, we hypothesize that ACE2‐EPC‐EXs might have better efficacy than EPC‐EXs in protecting ECs from H/R‐induced injury and oxidative stress. As expected, the data show that ACE2‐EPC‐EXs are more effective than EPC‐EXs in decreasing apoptosis, ROS overproduction and increasing EC function through down‐regulating Nox2 and up‐regulating eNOS.

Of note, based on our data of ACE2‐EPC‐EXs co‐culture, the differences of the ROS/Nox2/apoptosis decline and eNOS/NO/tube formation elevation appeared to be much more in Ang II‐induced ageing ECs than that of young ECs underwent H/R injury. The mechanism might be at least partially attributed by the higher level of ACE2 in ageing ECs after co‐incubation with ACE2‐EPC‐EXs. These observations are supported by our previous study showing that ACE2 could protect brain from ischaemic injury with a tendency of age‐dependence 6. It is also supported by other studies showing that Nox inhibition could counteract oxidative stress in pulmonary and kidney arteries of aged rats, as well as in lungs of aged mice 32, 33, 34. The other mechanism could be related with their carried miRs. As mentioned above, stem cell‐derived EXs carry signal molecules such as miRs which are important regulators of a variety of cellular processes including cell survival and proliferation 35, 36. In this study, we found that ACE2 transfection in EPCs leads to an up‐regulation of miR‐18a in EPCs and EPC‐EXs as well as in ECs after co‐incubation. These data suggest that miR‐18a might be the key player mediating the enhanced protective effects of ACE2‐EPC‐EXs in ageing ECs under H/R condition. To investigate the role of miR‐18a in ACE2‐EPC‐EXs, we knocked down its expression in the parent cells, ACE2‐EPCs. We confirmed that the level of miR‐18a in ACE2‐EPC‐EXs is down‐regulated. More importantly, we found that the protective effects in ACE2‐EPCs‐EXs^anti‐miR‐18a^ group were dramatically decreased, indicating that miR‐18a participates in the protection effects elicited by ACE2‐EPC‐EXs. In addition, to confirm whether ACE2 could affect miR‐18a expression, we used ACE2‐specific inhibitor, DX600. We found that DX600 blocked ACE2‐induced increase in miR‐18a in EPCs, the EPC‐EXs and the target ECs after co‐incubation. More importantly, we identified that the changes of miR‐18a level were associated with the EC functions. DX600 or anti‐miR‐18a partially blocked the beneficial effects of ACE2‐EPC‐EXs on H/R‐injured ECs. These data further confirmed that ACE2 promoted the expression of miR‐18a and ACE2‐EPC‐EXs protect ECs from H/R‐induced dysfunction through ACE2, EPC‐EXs and miR‐18a.

Taken together, ACE2‐EPC‐EXs exhibited greater anti‐oxidative and anti‐apoptosis effects on ageing ECs than on young ECs undergo H/R injury through their carried miR‐18a and subsequently down‐regulation of the Nox2/ROS pathway. Further experiments may be also applied to examine the protective roles of ACE2‐EPC‐EXs *in vivo* and to determine the targets of miR‐18a. These approaches may greatly improve our understandings of the molecular basis of ACE2‐mediated protection against ischaemic stroke and may shed light on development of novel therapies.

## Conflict of interest

There is no conflict of interests.
